# Exploring Morphine-Triggered PKC-Targets and Their Interaction with Signaling Pathways Leading to Pain via TrkA

**DOI:** 10.3390/proteomes6040039

**Published:** 2018-10-06

**Authors:** Darlene A. Pena, Mariana Lemos Duarte, Dimitrius T. Pramio, Lakshmi A. Devi, Deborah Schechtman

**Affiliations:** 1 Department of Biochemistry, Chemistry Institute, University of São Paulo, Sao Paulo 05508-220, Brazil; darlenebqi@yahoo.com.br (D.A.P.); dimibiomed@hotmail.com (D.T.P.); 2 Department of Pharmacological Sciences, Icahn School of Medicine at Mount Sinai, New York, NY 10029, USA; mlduarte@gmail.com

**Keywords:** morphine, opioid receptors, conformational antibody, analgesia, GPCR signaling

## Abstract

It is well accepted that treatment of chronic pain with morphine leads to μ opioid receptor (MOR) desensitization and the development of morphine tolerance. MOR activation by the selective peptide agonist, D-Ala2, N-MePhe4, Gly-ol]-enkephalin(DAMGO), leads to robust G protein receptor kinase activation, β-arrestin recruitment, and subsequent receptor endocytosis, which does not occur in an activation by morphine. However, MOR activation by morphine induces receptor desensitization, in a Protein kinase C (PKC) dependent manner. PKC inhibitors have been reported to decrease receptor desensitization, reduce opiate tolerance, and increase analgesia. However, the exact role of PKC in these processes is not clearly delineated. The difficulties in establishing a particular role for PKC have been, in part, due to the lack of reagents that allow the selective identification of PKC targets. Recently, we generated a conformation state-specific anti-PKC antibody that preferentially recognizes the active state of this kinase. Using this antibody to selectively isolate PKC substrates and a proteomics strategy to establish the identity of the proteins, we examined the effect of morphine treatment on the PKC targets. We found an enhanced interaction of a number of proteins with active PKC, in the presence of morphine. In this article, we discuss the role of these proteins in PKC-mediated MOR desensitization and analgesia. In addition, we posit a role for some of these proteins in mediating pain by TrKA activation, via the activation of transient receptor potential cation channel subfamily V member 1 (TRPV1). Finally, we discuss how these new PKC interacting proteins and pathways could be targeted for the treatment of pain.

## 1. Introduction

### Morphine-Mediated Signal Transduction Pathways and Receptor Desensitization

Treatment of chronic pain has been a challenge as the most effective treatment that uses opiates has many unwanted side effects; for example, chronic exposure leads to desensitization of opioid receptors, development of tolerance, and addiction [1]. One of the alarming effects, reported in 2016, is that more than 100 people die daily due to opioid-related overdose (CDC/NCHS, National Vital Statistics System, Mortality, CDC Wonder, Atlanta, GA: US Department of Health and Human Services, CDC; 2017).

Opiates, such as morphine and heroin, interact with opioid receptors and it is generally thought that they function primarily via the activation of the μ opioid receptor (MOR), although, at high concentrations, they can activate δ and κ opioid receptors [2]. Opioid receptors are located both pre-and-post-synaptically and are coupled to the Gi/Go proteins. Upon ligand binding, Gi/Go-coupled receptors, acutely inhibit adenylyl cyclase (AC) activity, decreasing the levels of the cyclic AMP (cAMP) and decreasing the activity of the protein kinase A (PKA) [3], or of the exchange protein directly activated by cAMP (EPAC) [4]. Opioid receptor activation also leads to the stimulation of inward rectifying potassium channels and the inhibition of voltage-gated calcium channels, causing a decreased neurotransmitter release from the pre-synaptic nerve terminal. Thus, the net effect of acute opiate administration is to inhibit neuronal transmission, and this is thought to lead to analgesia [5].

Chronic opiate administration, on the other hand, has been shown to upregulate the activity of AC and PKA [6]. This upregulation of the cAMP pathway has been reported to occur in several regions of the brain, reduce analgesia, and is thought to contribute to opiate addiction [7]. 

In addition to PKA, opioid receptors have been shown to regulate a number of other kinases. Activation of opioid receptors leads to the activation of G protein-coupled receptor kinases (GRK), mitogen-activated protein kinase (MAPK), protein kinase B (PKB/AKT), calcium/calmodulin-dependent kinase II (CAMKII), and protein kinase C (PKC) [2,8,9,10,11,12,13]. Some of these kinases are thought to play a role in opiate-mediated tolerance, dependence [14], and addiction [15]. In the case of PKC, studies show that PKC inhibitors decrease receptor desensitization, development of opiate tolerance, and opiate addiction [16,17], (reviewed in [18]).

PKC is a family of serine/threonine kinases, composed of eleven different isoenzymes, divided into three sub-families. These include, (i) classical PKCs (cPKCs) including α, Βi, βII, and γ, which are calcium-dependent and are activated by phosphatidyl serine (PS) and diacylglycerol (DAG), (ii) novel PKCs including δ, ε, η, and θ, which are calcium-independent, but depend on PS and DAG for their activation, and (iii) atypical PKCs including ζ and λ/ι, which are calcium-independent [19] and are thought to be activated by protein–protein interactions [20]. Different PKC isoenzymes are expressed at different subcellular locations. For example, PKCα is found in both pre-and-post-synaptic sites, at the outer surface of synaptic vessels. However, PKCγ in adult rats is only expressed in postsynaptic dendrites, perikaryal cytoplasm, and postsynaptic densities. On the other hand, PKCε is found only in small and medium-sized dorsal root ganglion (DRG) neuronal soma, and presynaptic terminals of nociceptive neurons in the dorsal spinal horn (reviewed in [18]). It has not yet been determined if these PKCs are active, and what proteins they are interacting with, or are being phosphorylated by them.

Distinct PKC isoenzymes have been implicated in opioid receptor desensitization and addiction. Protein levels and activity of PKCα and γ are increased in the dorsal spinal cord, during chronic exposure to morphine [21,22]. Selective inhibitors for PKCα, γ, and ε completely reverse morphine-tolerance [18]. In particular, PKCγ has been suggested to play a central role in morphine tolerance, both in the spinal cord and the nucleus accumbens (NAc), having a role in sensory signal processing [23]. Determining the exact role of PKC, in addiction, has been difficult, due to the fact that PKC also plays a critical role in the formation and maintenance of memory [24,25], including drug-induced memory [26]. However, it is not clear how PKC is activated, following the activation of MOR, by morphine. Following are the possible mechanisms that follow from the MOR activation:

(i) MOR activation enables the Gβγ subunit to activate PLC which then would lead to PKC activation [27],
(ii) MOR activation leads to an activation of a Gq-coupled receptor that, in turn, leads to PLCβ activation, as seen in the case of M3 muscarinic receptor activation-mediated increase in the MOR desensitization [28].
(iii) MOR activation leads to activation of the receptor-coupled and non-coupled tyrosine kinases, which in turn lead to PLCγ activation.
(iv) MOR activation leads to the activation of a small G protein which would then activate PLCε and subtypes of PLCβ and γ [29].
(v) MOR activation leads to activation of PI3K which then activates PKC.

However, this last scenario has been shown to occur only in the case of atypical PKCs that are insensitive to DAG [30]. In intestinal epithelial cells, MOR has been shown to activate PI3K, via Gβγ, leading to a decrease in cell death [31]). Not much is also known about which PKC isoenzymes are activated by morphine. A recent study with DRG neurons and HEK-293 cells that were overexpressing MOR, showed that both PKCα and ε were activated at the plasma membrane within the first minute of the receptor activation by morphine, and that this activation was sustained for at least 20 min. This was specific to morphine, since MOR activation by DAMGO did not activate PKC, in this time frame. The authors also demonstrated that PKCα was activated by Gβγ, and led to MOR phosphorylation at specific sites, that restricted the plasma membrane localization of MOR and inhibited subsequent nuclear activation of the extracellular signal-regulated kinase (ERK) [32]. Sequential activation of PKCα and ε has been previously shown to be responsible for sustained ERK1/2 activation, upon mechanical stress [33]. If this also happens in the case of MOR activation by morphine, or whether both PKCs are activated simultaneously, remains to be determined. Therefore, understanding the spatial and temporal dynamics of the PKC signaling can help us elucidate the mechanisms that lead to MOR desensitization, mediated by these kinases.

It is clear that PKC has an important role not only in receptor desensitization but also in inhibition of receptor recycling [18,32]. One of the main features that distinguishes morphine from other potent MOR agonists, such as DAMGO and fentanyl, is that morphine activates PKC signaling (and minimally activates GRK), whereas, DAMGO robustly activates GRK which phosphorylates the receptor and recruits β-arrestin, leading to a receptor endocytosis and recycling [32]. Even though PKC has been demonstrated to be a key target in morphine-mediated receptor desensitization, the mechanism by which PKC mediates this process is still not clear. Targeting PKC itself to decrease receptor desensitization could be problematic, as PKC is involved in several processes, including mediating immunological responses specifically against viral infections [34]. Thus, identifying PKC targets can be useful in elucidating the signal transduction processes involved in MOR desensitization and opioid-tolerance. MOR itself is a PKC target [35]. The carboxy-terminal tail of MOR contains 12 serine/threonine residues and two of them have the consensus sequence for phosphorylation by PKC. Mutations of eleven of these phosphorylation sites (including the two PKC sites) led to a functional receptor that was not desensitized or internalized, indicating that phosphorylation of MOR is important for receptor recycling [36,37]. A possibility that MOR activation of PKC leads to the phosphorylation of proteins other than the receptor, and that these PKC targets participate in desensitization, has not been well explored [35,38,39]. In order to address this issue, we developed a new strategy to identify the PKC interacting proteins/substrates within the context of an acute morphine treatment. For this, we used an antibody that specifically recognizes the active state of cPKCs (anti-C2Cat) [40]. Using this anti-C2Cat antibody, we immunoprecipitated active PKC-associated proteins from Neuro-2A cells treated with acute morphine, and identified the associated proteins by mass spectrometry. A number of proteins were identified, including a few known PKC targets. 

**Figure 1:**
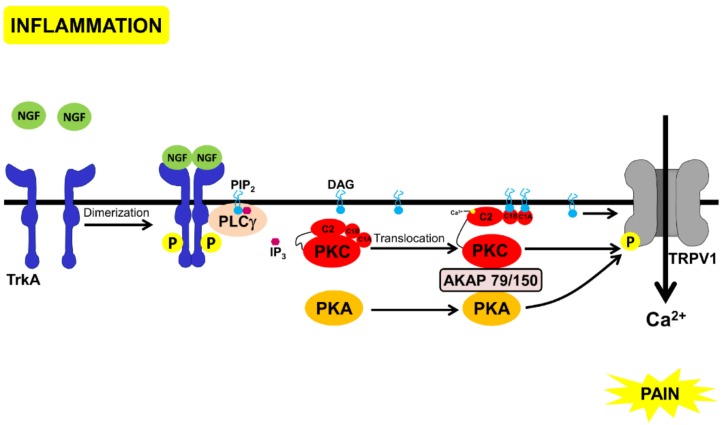
Schematic representation of the pathways that lead to pain through NGF-binding to TrkA. Inflammation leads to the secretion of NGF that binds to TrkA, leading to receptor dimerization and auto-phosphorylation, followed by PLCγ binding and activation. PLCγ cleaves inositol bisphosphate (PtdIns(4,5)P2) to inositol (1,4,5) trisphosphate and DAG. DAG directly activates TRPV1 [43] and the complex of PKC, PKA, and AKAP 79/150 that phosphorylates and also activates TRPV1 [42,44]).

In this article, we describe these proteins, discussing them in the context of pain mediated by nerve growth factor (NGF) signaling. In nociceptive neurons, NGF has a central role in pain. Inflammation leads to the release of NGF and activation of tropomyosin receptor kinase A (TrkA), a tyrosine kinase coupled to the NGF receptor [41]. NGF-binding leads to TrkA dimerization, auto-phosphorylation and subsequent binding and activation of PLCγ [42]. Amongst the several pathways activated by TrkA, PLCγ activation causes DAG generation and opening of an ion channel, the transient receptor potential cation channel subfamily V member 1 (TRPV1), a nonselective cation channel involved in a variety of nociceptive processes and activated by several stimuli (including acidic pH, heat, endocannabinoids, endogenous lipids, and capsaicin). Activation of TRPV1 causes a cation influx followed by depolarization and pain [43]. PKA and PKC bind to AKAP79/150, and this complex can then phosphorylate and activate TRPV1 [44] (Figure 1). One of morphine’s targets is TRPV1 (reviewed in [45]). Blocking PKA-signaling by MOR, inhibits the TRPV1 channel activity and the TRPV1 active multimer-translocation to the membrane [46]. Moreover, a cAMP analog, 8Br-cAMP, can reverse the opioid-mediated inhibition of TRPV1, in DRG neurons [47]. Furthermore, blocking TRPV1 decreases morphine tolerance [48]. In DRG neurons and the spinal cord, TRPV1 and MOR are co-localized and their expression increases, upon inflammation [48,49]. These observations suggest that TRPV1 [50] and TrkA [51,52] could be drug targets for the development of non-opioid analgesics. Therefore, understanding the interaction between morphine-mediated analgesia and TrkA mediated pain could lead to the development of analgesics with lesser side-effects than the currently used drugs.

## 2. Methods

### 2.1. Cell Culture

Mouse neuroblastoma (Neuro 2A) cell line was obtained from American Type Culture Collection (Manassas, VA, USA) and maintained in DMEM high Glucose, supplemented with 10% fetal bovine serum (FBS), penicillin/streptomycin [50 U/mL and 50 μg/mL, respectively, (Gibco-BRL^®^)], at 37 °C, under 5% CO_2_. 

### 2.2. Immunofluorescence

Neuro 2A cells were cultured on 13 mm glass coverslips, at 60% confluency, and treated with 50 nM PMA or 1 μM of Morphine or ATP for 1, 3, and 30 min. Cells were then fixed with 4% PFA, permeabilized with Phosphate buffered saline (PBS), 0.1% Triton X-100. Next, the cells were blocked in PBS, 0.1% Triton-X100, 1% normal goat serum, for 40 min, at room temperature. Cells were subsequently incubated overnight at 4 °C, with anti-C2Cat serum [40], diluted 1:100 in blocking solution, or anti PKCα (4 μg/mL, Santa Cruz Biotechnology^®^) and incubated for 1 h, at room temperature (RT), with anti-rabbit conjugated to Alexa 555 (4 μg/mL), diluted as above. As a negative control, secondary antibodies were incubated alone (without prior incubation with primary antibodies) to assess the nonspecific-binding. Coverslips were mounted with Vectashield^®^/DAPI, and Immunofluorescence staining was analyzed using a Leica DM6000 fluorescent microscope. The level of fluorescence intensity from 12-15 images, for each condition, with an average of 60 cells per field, was quantified using ImageJ^®^ software and the amount of cPKC activity in each treatment was normalized to the control levels (fluorescence detected in unstimulated cells), which was set to 100. Statistical analyses of ANOVA (Dunnett’s test) was done.

### 2.3. Western Blot

To analyze the cPKC expression in Neuro2A cells, cells were cultured in 25-cm^2^ flasks in DMEM high Glucose, supplemented with FBS and antibiotics, until they reached 60% to 80% confluence. The total cells were counted using a Neubauer chamber, to get 1 × 10^6^ cells. The cells were then lysed in Laemmli buffer, run on 10% SDS-PAGE, and the separated polypeptide chains were transferred to nitrocellulose membranes, as described previously [40]. Membranes were incubated for 2 h, at RT, with anti-PKCα, βI, βII or γ (0.4 μg/mL, Santa Cruz Biotechnology^®^), and anti-α-tubulin (1:5000, Sigma-Aldrich^®^) as primary antibodies diluted in PBS/0.1% Tween-20, and 10% non-fat milk. Goat secondary antibodies, anti-rabbit IgG, and anti-mouse IgG, conjugated to horseradish peroxidase (GE Healthcare Life Science^®^), were diluted 1:1000 in PBS/0.1% Tween-20. For the negative control, membranes were incubated with the secondary antibodies only (without prior incubation with primary antibodies). Immunodetection was performed by chemiluminescence and the quantification was performed using ImageJ^®^ software.

### 2.4. Real-Time PCR

Total RNA was extracted from 1 × 10^6^ Neuro 2A cells with TRIzol^®^ (ThermoFisher Scientific, Waltham, MA, USA), following the manufacturer’s instructions. cDNA was synthesized with iScript cDNA synthesis kit (Bio-Rad^®^, Hercules, CA, USA). Real-time PCR reactions were performed with the PowerUp SYBR^®^ Green Master Mix (ThermoFisher Scientific) on a 7500 Real-Time PCR System (Applied Biosystems, Foster City, CA, USA), using the reaction default parameters. Primers used on the reactions were the following: PKCα Fwd: 5′CTGGAGAACAGGGAGATCCA3′, Rev: 5′ ACTGGGGGTTGACATACGAG3′; PKCβI Fwd: 5′AGAAACTCGAACGCAAGGAG3′, Rev: 5′CGAGAAGCCAGCAAACTCAT3′; PKCβII Fwd: 5′ AGAAACTCGAACGCAAGGAG3′, Rev: 5′TCCTGATGACTTCCTGGTCA3′; PKCγ Fwd: 5′GGAAATTGCACCTCCTTTCA3′, Rev: 5′ACGAAGTCCGGGTTCACATA3′; GAPDH Fwd: 5′AGGTCGGTGTGAACGGATTTG3′, Rev: 5′GGGGTCGTTGATGGCAACA3′. Relative abundance of the transcripts was normalized by the expression of the *GAPDH* gene and calculated with the equation: 2^20^ − [Ct(PKC) − Ct(GAPDH)], where 2^20^ would be an arbitrary number of copies of GAPDH [53]. 

### 2.5. Cross-Linking Antibodies to Beads

The antibodies anti-C2Cat (5 μL) or pre-immune serum (5 μL) [40] were crosslinked to protein G beads [(50 μL) (Invitrogen^®^)], for use in immunoprecipitation assays. Briefly, beads were washed, by centrifugation, with 1 mg/mL BSA, in PBS. Anti-C2Cat (5 μL) or pre-immune serum (5 μL) were incubated with 50 μL of packed volume of protein G beads, for 1 h at 4 °C. Antibody-bound beads were washed with 1 mg/mL BSA, in PBS, and resuspended in PBS. Beads were incubated under rotation with dimethylpimelimidate [(DMP) (Sigma^®^)] solution, freshly made up in 0.2 M triethanolamine, with the pH readjusted to pH 8.2, for 30 min at RT. Subsequently, beads were washed with 0.2 M of triethanolamine, in PBS, by incubation for 5 min, at RT, under rotation and then incubated twice with an equal volume of DMP solution, for 5 min at RT, under rotation. The reaction was stopped by an addition of an equal volume of 50 mM ethanolamine, prepared in PBS. After 5 min of incubation at RT under rotation, the excess (non-bounded) antibody was removed by washing with 1M glycine pH 3.0, and beads were resuspended in a PBS buffer.

### 2.6. Immunoprecipitation Assays and iTRAQ^®^

For the immunoprecipitation assays, Neuro 2A cells were cultured in 75-cm^2^ flasks, in DMEM high Glucose supplemented with FBS and antibiotics, until they reached 60% to 80% confluence (containing approximately 5 × 10^6^ cells). Neuro 2A cells were then treated with either saline or morphine (1 μM), for 3 min, and lysed in PBS containing 1% Triton X-100, protease (Sigma-Aldrich^®^), and phosphatase [PhosphoStop™ (Sigma^®^)] inhibitor cocktails, followed by three freeze–thaw cycles. Cells were sonicated for 30 min, at 80 Hz (output), with a probe sonicator (Branson Sonifier 250). Cell lysates were precleared with protein G beads, for 1 h at 4 °C, incubated with crosslinked antibody-bound beads, overnight at 4 °C, and subsequently washed with PBS. Experimental triplicates of immunoprecipitated proteins with anti-C2Cat from control (vehicle-treated) or morphine-treated cells were performed and individual samples from the cells immunoprecipitated with pre-immune serum, in the presence or absence of morphine, were prepared, totaling 8 samples. Proteins were eluted with 0.1 M glycine pH 3.0 and subject to SDS-PAGE, followed by in-gel trypsin digestion. The resulting peptides were subjected to 8-plex iTRAQ^®^ (isobaric tags for relative and absolute quantitation), essentially, as previously described [54]. Labeling was performed according to the manufacturer’s protocol, using the following isobaric iTRAQ^®^ tags: 113, 114, and 115 for saline-treated samples, immunoprecipitated with anti-C2Cat, 116, 117, and 118 for morphine-treated samples, immunoprecipitated with anti-C2Cat, 119, and 121 for saline and morphine, respectively, both immunoprecipitated with pre-immune serum. After that, the iTRAQ^®^ labeled peptides from saline and morphine-treated samples were combined and subjected to strong cation exchange liquid chromatography (SCXLC). Fractions containing the labeled peptides were analyzed by RPLC-MS/MS on Obitrap Velos mass spectrometer. MS/MS spectra were searched against UniRef 100 mouse database, using Mascot search engine. A total of 2889 unique peptides, corresponding to 956 proteins, were identified. Homologous protein redundancy was reduced by Scaffold software (http://www.proteomesoftware.com/QPlus/ScaffoldQ+.html) to a minimum. The false discovery rate was less than 1%. Each protein was identified with at least one unique peptide, with FDR less than 1%.

To identify the proteins that preferably interacted more with cPKC, upon morphine treatment, proteins were analyzed using the Scaffold software, as discussed in the results section and Figure 3D. Further network analysis of PKC interaction proteins was performed using Strings (https://string-db.org/) webtool. Immunoprecipitated proteins that interacted more with anti-C2Cat, in the presence of morphine and PKCα, were analyzed for their interaction with each other, according to the following criteria. (i) text mining, (ii) experiments, and (iii) databases. The analysis was performed with a minimum confidence of 0.4. Seventeen proteins were shown to interact with PKCα, according to these criteria, and a network of the interaction amongst these proteins was made. The analysis was performed with a minimum confidence of 0.4.

## 3. Results

Previously, we have developed and characterized an antibody that preferentially recognizes active cPKC. This antibody was used to study the spatial and temporal dynamics of active cPKC, in SK-N-SH cells, activated by the DAG analog, Phorbol myristate acetate (PMA), or activated by ATP and glutamate [40]. The anti-active state cPKC antibody was named anti-C2Cat, since this antibody is directed towards an intramolecular interaction between the C2 and the catalytic domains of PKC that occurs only in an inactive kinase, and thus the epitope recognized by the antibody was only exposed upon activation [40]. Previously, we have used this antibody to immunoprecipitate cPKC from breast cancer cell lines and found higher levels of active cPKC in a metastatic breast cancer cell line (MDA-MB-231), compared to a non-metastatic cell line (MCF-7). We also, showed a significantly higher level of active cPKC in metastatic, triple negative breast cancer samples, as compared to estrogen receptor positive (ER+) samples [40].

**Figure 2:**
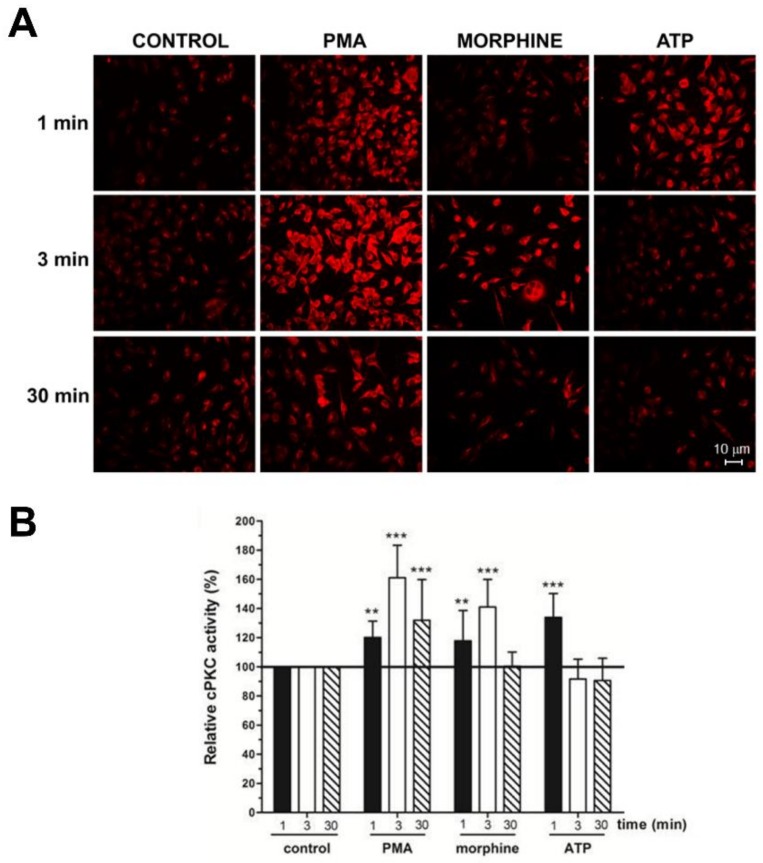
Morphine and Phorbol ester triggered cPKC activation with anti-C2Cat. (**A**) Neuro-2A cells were treated with 1 μM Morphine, ATP, or 100 nM PMA, for the indicated periods. Activators were removed by washing with PBS and the cells were fixed for immunofluorescence analysis. cPKC activation was performed using anti-C2Cat [40] as the primary antibody, and as a secondary antibody, when conjugated with Alexa 568. (**B**) The level of fluorescence intensity was quantified using ImageJ^®^ software and the amount of cPKC activity, in each treatment, was normalized to the control levels (fluorescence detected in unstimulated cells), which was set to 100. Results represent the average ± SD of measurements from twelve to fifteen different images, for each condition, statistical significance was determined by ANOVA–Dunnett’s test where *** represents *p* < 0.001 and ** represents *p* < 0.01.

**Figure 3:**
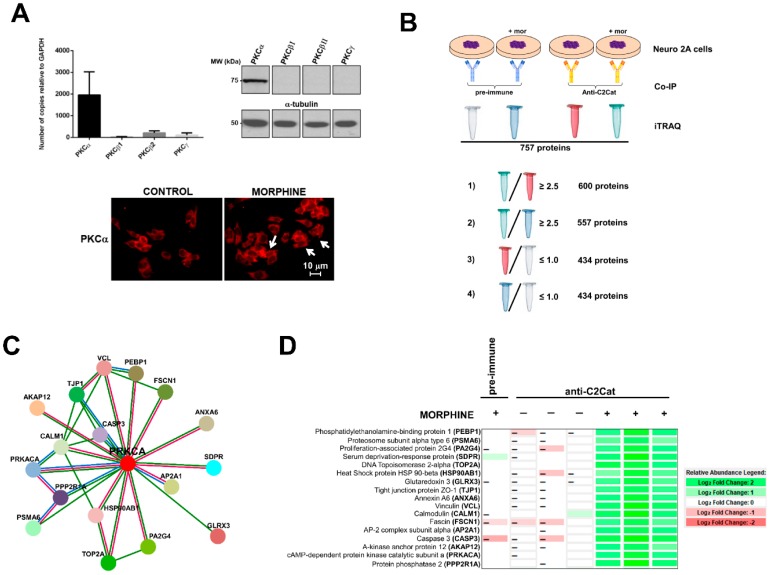
cPKC binding partners found in Neuro 2A cells that interacted more with anti-C2Cat, upon treatment with 1 μM morphine, for 3 min. (**A**) cPKC expression in Neuro2A cells analyzed by Real-time PCR (average of *n* = 3), and Western blot, with isoenzyme specific antibodies (top panels), immunofluorescence of PKCα, in control or morphine-treated cells (1 μM morphine for 3 min), white arrows indicate that PKCα was present in the membrane (indicative of PKC activation). (**B**) A representative diagram of how the proteins that interacted more with anti-C2Cat, upon morphine activation of MOR, were selected to eliminate non-specific binding (as discussed in the results section). (**C**) Using Strings (https://string-db.org/), a network with 17 PKCα interacting proteins was created (as discussed in the text). (1) Red lines indicate PKCα interacting proteins obtained from both experimental data and text mining (proteins that were co-mentioned). (2) Green lines indicate proteins that were co-mentioned. (3) Blue lines indicate putative homologs co-mentioned in other species. (**D**) A Heat map indicating a quantitative analysis of the intensities of the peptides found for each of the 17 proteins, which were found to interact more with anti-C2Cat, in the presence of morphine.

In this study, we used anti-C2Cat to study the temporal dynamics of PKC activation, after stimulation of Neuro 2A with morphine, ATP, and PMA. As can be seen in Figure 2, PKC was active after 3 min of treatment, with 1 μM morphine, and its activation was faster (1 min) when ATP was used. Treatment with PMA led to the PKC activation, by 1 min, and this was sustained for up to 30 min, after treatment. Of the classical PKCs, PKCα was expressed the most, in Neuro2A cells, as seen in Figure 3A. cPKCs were detected, both, by real-time PCR and Western blot with isoenzyme specific antibodies. Immunofluorescence with anti-PKCα antibodies, following treatment with 1 μM morphine, for 3 min, detected an increase in PKCα at the cell membrane (indicative of active PKC) (Figure 3A, bottom).

To detect proteins and substrates with enhanced interaction with PKC, in response to MOR activation by morphine, immunoprecipitation experiments with anti-C2Cat (and pre-immune serum as a control) were performed with Neuro 2A cells, treated with and without 1μM morphine, for 3 min (Figure 3B). Immunoprecipitated proteins were labeled by iTRAQ^®^ and submitted to mass spectrometry, as described in Methods. We identified 757 proteins, excluding putative and uncharacterized proteins. Based on the quantitative data of the immunoprecipitated proteins (analyzed using Scaffold 4 software), the following criteria were used to select proteins that exhibited enhanced interaction with PKC, in response to MOR activation by morphine:

(i) To detect the proteins that interacted with active PKC upon morphine treatment, we eliminated proteins that immunoprecipitated from cells not treated with morphine. Six hundred proteins met this criterion (*p* ≤ 0.05, per t-test analysis) and these proteins exhibited a ratio of ≥2.5, when proteins immunoprecipitated from morphine-treated cells were compared with vehicle-treated cells.
(ii) To exclude proteins that bound non-specifically to the anti-C2Cat antibody, we eliminated proteins that interacted with pre-immune serum in the presence of morphine. This reduced the list to 557 proteins and these proteins exhibited a ratio ≥2.5, when proteins that were immunoprecipitated by anti-C2Cat antibody were compared to proteins immunoprecipitated with pre-immune serum (from cells treated with morphine).
(iii) To exclude proteins that interacted with PKC under basal conditions (absence of morphine), we only considered proteins that had a ratio ≤1.0, when comparing proteins immunoprecipitated with anti-C2Cat, to those with pre-immune serum, in vehicle-treated cells. This further reduced the list to 434 proteins.

Of these, approximately 20% of the identified proteins were ribosomal proteins or proteins involved with RNA processing and 20% involved in metabolism. We did not immunoprecipitate PKC, because of limitations of mass spectrometry. However, when the immunoprecipitated proteins were subjected to Western Blotting, using an anti-PKCα antibody, we found higher levels of PKCα signal in the anti- C2Cat immunoprecipitate of cells treated with morphine (Supplementary Figure S1). Next, we used Strings (https://string-db.org/) software to generate a network of the anti-C2Cat immunoprecipitated proteins, from cells treated with morphine; of these seventeen proteins were shown to directly interact with PKCα (Figure 3C). As can be seen in this figure some proteins interacted with each other, suggesting they could be in a complex of PKCα and associated binding proteins (Figure 3D). Other proteins in the list also interacted with the known PKCα-binding proteins previously identified, for example, eleven proteins have been previously reported to interact with the catalytic domain of PKA (PRKACA), including the regulatory domain. Below we discuss the role for some of these proteins in the context of the pain-signaling pathway, activated by the Nerve Growth Factor (NGF) and the Tropomyosin Receptor Kinase A (TrkA).

A heat map shown in Figure 3D summarizes the relative quantification of the 17 proteins immunoprecipitated with anti-C2Cat, from cells treated with morphine (and compares it to those immunoprecipitated following vehicle treatment or immunoprecipitated with pre-immune serum from morphine-treated cells). Data were analyzed using Scaffold 4 (Proteome Software, Inc.). It was clearly seen that these 17 proteins interacted more with anti-C2Cat antibody i.e., active cPKC, upon MOR stimulation with morphine, and that our strategy with activation-specific antibodies enriched the PKC binding partners (Figure 3D).

Below, we discuss some of the identified PKCα interacting proteins, detected with the anti-active-state specific cPKC antibody and a PKC substrate (neurogranin), previously validated in the literature [55], that could be regulating spatial and temporal dynamics, in the context of TrkA and MOR signaling.

### 3.1. Phosphatidylethanolamine Binding Protein 1 (PEBP1)

**Figure 4:**
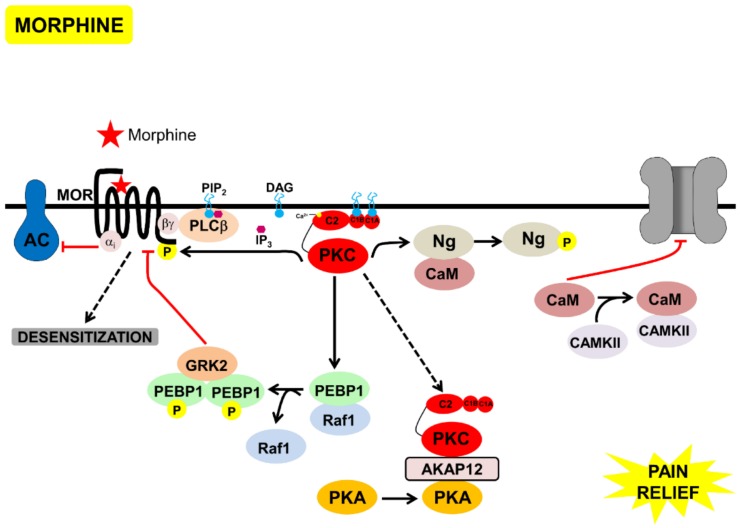
Model of the signaling processes mediated by acute morphine, that lead to desensitization of MOR and pain-relief, through inhibition of TRPV1. Morphine bound to MOR, leading to Gαi-mediated inhibition of AC and Gbγ-mediated activation of PKC. PKC phosphorylated neurogranin bound to calmodulin, calmodulin was then released and inhibited TRPV1. PKC phosphorylated PEBP1, releasing it from Raf1. Phosphorylated PEBP1 dimerized, bound to, and inhibited GRK2, inhibiting β-arrestin-mediated MOR endocytosis, leading to MOR desensitization. Active PKC bound to AKAP12, leading to PKA relocalization to the cytoplasm, and inhibition of TRPV1 phosphorylation by PKC/PKA.

As discussed above, one of the main features that distinguish DAMGO from morphine is that MOR activated by DAMGO leads to robust GRK activation, while MOR activated by morphine activates PKC signaling, without significant GRK activation [32]. One of the mechanisms for this could be through the Phosphatidylethanolamine-binding protein 1 (PEBP1) phosphorylation by PKC. PEBP1, also known as Raf kinase inhibitory protein (RKIP), which inhibits Raf1-signaling and thus the MAPK activation. PKC phosphorylates Serine 153 of PEBP1, leading to its dimerization, as dimerized PEBP1 is known to interact with and inhibit GRK2 [56] (Figure 4). GRK2 phosphorylation of MOR was essential for arrestin-binding and endocytosis [18,57]. Interestingly, non-phosphorylated PEBP1 has also been shown to bind to δ-opioid receptors (DOR), and inhibit Gαβγ binding to DOR. Upon PKC activation, phosphorylated PEBP1 binds to GRK2, enabling G αβγ-binding and signaling, via DOR [58].

### 3.2. Scaffolds (Annexin 6 and AKAP12) and PKA

Spatial dynamics of signaling pathways is essential to position proteins at specific subcellular localizations. Adaptor proteins often mediate interactions between kinases and substrates, and help create microdomains. Of these, we found two scaffolds that have been shown to interact with PKC, annexin 6 [59], and AKAP12, also known as, Gravin, SSeCKS, or AKAP250 [60].

The role of PKC activation/inhibition of ERK by PEBP1 and Annexin 6 should be more carefully examined. Annexin 6 binds Ras-GTPase activating protein p120GAP, leading to Ras-GDP and consequently inhibiting ERK activation, besides being a scaffold protein for PKC [61]. Recently, Halls and collaborators (2016) showed that the spatial and temporal dynamics of ERK-activation is regulated by morphine versus DAMGO. They also showed that PKCα is a key player for this regulation, since it inhibits the receptor translocation within the plasma membrane which triggers a transient nuclear activation of the ERK, while sustaining cytosolic ERK-activation [32]. This study suggests that modulators of ERK-signaling, such as PEBP1 and Annexin 6, should be analyzed within the context of spatial-signaling, triggered by morphine.

AKAP12 is a PKA substrate and scaffold [62] that has been shown to interact with a β-adrenergic receptor and mediate its phosphorylation and desensitization. AKAP12 is localized at the plasma membrane and relocalizes to the cytoplasm, upon an increase in calcium in a PKC-dependent manner [60]. Indeed, we found AKAP12 and PKA (both regulatory and catalytic domains), interacting with active PKC, upon morphine treatment. The redistribution of PKA, by AKAP12, to the cytoplasm, upon PKC activation, could be a mechanism to inhibit the PKA membrane localization and the binding of other AKAPS (such as 79/50). Whether AKAP12 interacts with PKC, at the membrane, and is phosphorylated and relocalized to the cytoplasm, or whether PKCα relocalizes with AKAP12, upon activation, remains to be investigated. Interestingly, both, PKC and PKA phosphorylate TRPV1, at specific residues, increasing the channel activity and decreasing the threshold for channel activation (reviewed by [63]). These phosphorylations only occur upon binding of the kinases, PKA and PKC, to AKAP79/150 [44]. It is possible that morphine could not only be inhibiting the PKA activity through Gαi, but could also be mediating PKA (regulatory and catalytic domains) redistribution via PKC, and this should be further investigated.

### 3.3. Neurogranin and Calmodulin

Neurogranin (Ng) is one of the few well-characterized PKC substrates, in the context of morphine treatment [55]. Neurogranin is expressed in post-synaptic neuronal cell bodies and dendrites of the hippocampus, amygdala, striatum, and olfactory bulb, and interacts both with Ca^2+^/calmodulin dependent protein kinase II (CAMKII) and PKC, and has been shown to be a regulator of CAMKII activity. At low levels of calcium, neurogranin binds Calmodulin (CaM), sequestering it and inhibiting it from binding to CAMKII. An increase in cytosolic calcium releases Ng from CaM, enabling it to bind to and activate CAMKII. Phosphorylation of neurogranin, by PKC, can also release CaM bound to Ng and lead to CAMKII activation. Free CaM can also bind directly to TRPV1, and prevent AKAP79/150 bound to PKC and PKA, from binding to TRPV1, consequently inhibiting the channel and causing a decrease in pain sensation (Figure 4).

An increase in neurogranin phosphorylation and in CaMKII activity was observed in the brains and spinal cord of opioid-tolerant mice [55]. Inhibition of neurogranin, through antisense oligodeoxynucleotides, decreased morphine dependence, and activation of CaMKII and of the transcription factor cAMP response element-binding protein (CREB) [64]. Further, neurogranin phosphorylation potentiates synaptic transmission and long-term potentiation (LTP) processes [65], which are important for the development of opioid tolerance. Neurogranin is an important link between PKC and CAMKII, which are two key proteins involved in the development of tolerance [55].

### 3.4. Morphine Inhibition of TrKA-Signaling Pathways via PKC

Signaling networks in the context of specific processes are complex and often involve cross-talk between different pathways and receptors. As discussed above, besides activation of PKC by Gβγ, in Gι coupled receptors, activation/sustained activation of PKC could possibly be mediated by subsequent activation of Gαq coupled receptors, tyrosine kinases, and small G proteins via PLC activation [28,29]

In the context of morphine signaling, we proposed a cross-talk between MOR, PKC, TrkA, and TRPV1, which is summarized below.

Upon inflammation, NGF is secreted and, via PLCγ, it activates TRPV1 leading to pain (Figure 1). On the other hand, attenuation of pain by acute morphine administration leads to the following:

(a) Inhibition of PKA by Gαi.
(b) Transient activation of cPKC, initially via Gβγ. Sustained PKC activation, possibly of nPKCs, could be mediated through other mechanisms discussed above.
(c) PKC-mediated phosphorylation of neurogranin, making CAM available to activate CAMKII and to bind to TRPV1, and inhibit the channel and nociception.
(d) Displacement of PKC and PKA, through scaffold proteins, annexin 6, and AKAP 12, inhibiting these kinases from binding to AKAP79/150 and activating TRPV1 (Figure 4).

In part, morphine-mediated desensitization could be due to the inhibition of endocytosis and receptor recycling via β-arrestin. Phosphorylation of PEBP1 by PKC leads to localized MAPK-activation and inactivation of GRK2, thereby, inhibiting MOR phosphorylation, internalization by β-arrestin, and receptor recycling. Upon chronic morphine treatment, PKA was activated [45] leading to an increase in NGF secretion [66]. PKC and PKA activation led to TRPV1 activation and pain, these mechanisms should be further explored in future studies. Other mechanisms of PKC activation upon chronic morphine exposure, such as EPAC-mediated PKCε activation, should also be explored [67]. MOR activation, in fact, has been shown to cause a decrease in EPAC activation [68]. 

## 4. Concluding Remarks

Despite the fact that PKC plays a role in MOR desensitization, the exact role and mechanisms that lead to receptor desensitization, via this kinase family, are still unclear. One of the reasons is the difficulty in identifying kinase interaction proteins. Using an active-state specific PKC antibody we identified proteins that interact with PKC upon MOR-activation and we discuss pathways that could be activated by these PKC interacting proteins, in the context of acute morphine treatment. Future studies will validate the proposed interactions. These proteins should be validated within the context of pain, in animal models.

Pain is discussed in the context of TrKA and TRPV1 activation, and its attenuation by morphine. Other cation channels involved in depolarization and pain could be affected in a similar manner. Due to the opioid epidemic, it is important to integrate signaling pathways and understand the molecular mechanisms involved in MOR desensitization, as this may lead to new strategies to modulate morphine-signaling and decreasing tolerance. Mutations in TrKA lead to *Congenital Insensitivity to Pain with Anhidrosis* (CIPA) [69,70], and a decrease in kinase activity observed in the naked mole rat leads to a reduction in pain sensitivity [71]. Efforts to develop selective inhibitors, for both TRPV1 [50] and TrKA [51,52], are underway. New and more specific therapeutic approaches towards pain can be developed, upon understanding the molecular mechanisms of these signaling pathways.

## Supplementary Materials

The following are available online at http://www.mdpi.com/2227-7382/6/4/39/s1, Figure S1: PKCα was immunoprecipitated with anti-C2Cat antibody following morphine treatment. Western blot probing for PKCα in two of the three samples processed for protein quantification and identification experiments by Mass Spectrometry, in the presence and absence of 1 μM Morphine for three minutes. No reactivity with anti PKCα was found in samples immunoprecipitated with Pre-immune serum (data not shown).
