# Supplementary material for: Exploring Morphine-Triggered PKC-Targets and Their Interaction with Signaling Pathways Leading to Pain via TrkA
Source: Proteomes. 2018 Oct 6;6(4):39. doi: 10.3390/proteomes6040039 (PMC6313901; doi:10.3390/proteomes6040039)
Supplement: Supplementary file 1 [file proteomes-06-00039-s001.pdf]

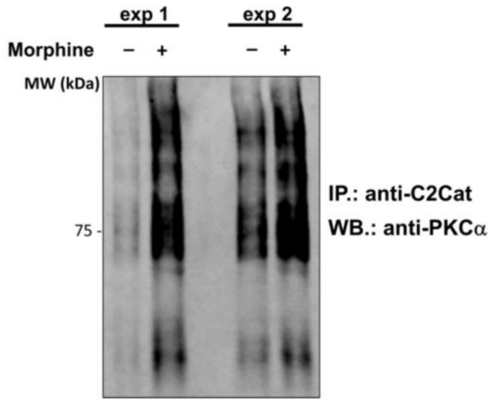

Figure S1. PKCα was immunoprecipitated with anti-C2Cat antibody following morphine treatment. Western blot probing for PKCα in two of the three samples processed for protein quantification and identification experiments by Mass Spectrometry, in the presence and absence of 1 μM Morphine for three minutes. No reactivity with anti PKCα was found in samples immunoprecipitated with Pre-immune serum (data not shown).
